# Parental Origin of Interstitial Duplications at 15q11.2-q13.3 in Schizophrenia and Neurodevelopmental Disorders

**DOI:** 10.1371/journal.pgen.1005993

**Published:** 2016-05-06

**Authors:** Anthony R. Isles, Andrés Ingason, Chelsea Lowther, James Walters, Micha Gawlick, Gerald Stöber, Elliott Rees, Joanna Martin, Rosie B. Little, Harry Potter, Lyudmila Georgieva, Lucilla Pizzo, Norio Ozaki, Branko Aleksic, Itaru Kushima, Masashi Ikeda, Nakao Iwata, Douglas F. Levinson, Pablo V. Gejman, Jianxin Shi, Alan R. Sanders, Jubao Duan, Joseph Willis, Sanjay Sisodiya, Gregory Costain, Thomas M. Werge, Franziska Degenhardt, Ina Giegling, Dan Rujescu, Stefan J. Hreidarsson, Evald Saemundsen, Joo Wook Ahn, Caroline Ogilvie, Santhosh D. Girirajan, Hreinn Stefansson, Kari Stefansson, Michael C. O’Donovan, Michael J. Owen, Anne Bassett, George Kirov

**Affiliations:** 1 Cardiff University, MRC Centre for Neuropsychiatric Genetics and Genomics, Institute of Psychological Medicine and Clinical Neurosciences, Cardiff University, Cardiff, United Kingdom; 2 DeCode Genetics, Reykjavik, Iceland; 3 Clinical Genetics Research Program, Centre for Addiction & Mental Health, University of Toronto, Toronto, Ontario, Canada; 4 University of Würzburg, Würzburg, Germany; 5 Oxford Gene Technology, Begbroke, Oxfordshire, United Kingdom; 6 Department of Biochemistry and Molecular Biology and Department of Anthropology, University Park, Pennsylvania, United States of America; 7 Department of Psychiatry, Nagoya University Graduate School of Medicine, Showa-ku, Nagoya City, Aichi, Japan; 8 Department of Psychiatry, Fujita Health University School of Medicine, Toyoake, Aichi, Japan; 9 Department of Psychiatry, Stanford University, Palo Alto, California, United States of America; 10 Department of Psychiatry and Behavioral Sciences, Stanford University, Stanford, California, United States of America; 11 Biostatistics Branch, Division of Cancer Epidemiology and Genetics, National Cancer Institute, Medical Center Drive, Bethesda, Maryland, United States of America; 12 Department of Psychiatry and Behavioral Sciences, NorthShore University HealthSystem, Evanston, Illinois, United States of America; Department of Psychiatry and Behavioral Neuroscience, University of Chicago, Chicago, Illinois, United States of America; 13 UCL Institute of Neurology, Queen Square, London, United Kingdom; 14 Institute of Biological Psychiatry, Mental Health Centre Sct. Hans, Mental Health Services Copenhagen, University of Copenhagen, Copenhagen, Denmark; 15 Institute of Human Genetics, University of Bonn, Bonn, Germany; 16 Department of Psychiatry, University of Halle, Halle, Germany; 17 The State Diagnostic and Counselling Centre, Kópavogur, Iceland; 18 Faculty of Medicine, University of Iceland, Reykjavík, Iceland; 19 Guy's and St Thomas' NHS Foundation Trust, London, United Kingdom; University of Pennsylvania, UNITED STATES

## Abstract

Duplications at 15q11.2-q13.3 overlapping the Prader-Willi/Angelman syndrome (PWS/AS) region have been associated with developmental delay (DD), autism spectrum disorder (ASD) and schizophrenia (SZ). Due to presence of imprinted genes within the region, the parental origin of these duplications may be key to the pathogenicity. Duplications of maternal origin are associated with disease, whereas the pathogenicity of paternal ones is unclear. To clarify the role of maternal and paternal duplications, we conducted the largest and most detailed study to date of parental origin of 15q11.2-q13.3 interstitial duplications in DD, ASD and SZ cohorts. We show, for the first time, that paternal duplications lead to an increased risk of developing DD/ASD/multiple congenital anomalies (MCA), but do not appear to increase risk for SZ. The importance of the epigenetic status of 15q11.2-q13.3 duplications was further underlined by analysis of a number of families, in which the duplication was paternally derived in the mother, who was unaffected, whereas her offspring, who inherited a maternally derived duplication, suffered from psychotic illness. Interestingly, the most consistent clinical characteristics of SZ patients with 15q11.2-q13.3 duplications were learning or developmental problems, found in 76% of carriers. Despite their lower pathogenicity, paternal duplications are less frequent in the general population with a general population prevalence of 0.0033% compared to 0.0069% for maternal duplications. This may be due to lower fecundity of male carriers and differential survival of embryos, something echoed in the findings that both types of duplications are *de novo* in just over 50% of cases. Isodicentric chromosome 15 (idic15) or interstitial triplications were not observed in SZ patients or in controls. Overall, this study refines the distinct roles of maternal and paternal interstitial duplications at 15q11.2-q13.3, underlining the critical importance of maternally expressed imprinted genes in the contribution of Copy Number Variants (CNVs) at this interval to the incidence of psychotic illness. This work will have tangible benefits for patients with 15q11.2-q13.3 duplications by aiding genetic counseling.

## Author Summary

The genetic interval 15q11.2-q13.3 on human chromosome 15 contains several so-called “imprinted genes” which are subject to epigenetic marking leading to activity from only one parental copy. This is in contrast to non-imprinted genes, whose activity is independent of their parent-of-origin. Deletions affecting the 15q11.2-q13.3 interval cause Prader-Willi and Angelman syndromes (PWS/AS), depending on whether the deletions are paternally or maternally derived respectively. Duplications at the PWS/AS interval region may also lead to neurodevelopmental disorders, including developmental delay (DD), autism spectrum disorder (ASD) and schizophrenia (SZ). Due to presence of imprinted genes within the region, the parental origin of these duplications may be key to the pathogenicity. We show, for the first time, that paternal duplications lead to an increased risk of developing DD/ASD/multiple congenital anomalies (MCA) but, unlike maternal duplication, do not appear to increase risk for SZ. This study refines the distinct roles of maternal and paternal duplications at 15q11.2-q13.3, underlining the critical importance of maternally active imprinted genes in the contribution to the incidence of psychotic illness. This work will have tangible benefits for patients with 15q11.2-q13.3 duplications by aiding genetic counseling.

## Introduction

Recurrent duplications of ~4Mb at 15q11.2-q13.3, overlapping the Prader-Willi syndrome /Angelman syndrome (PWS/AS) region between breakpoints 2 and 3 or 1 and 3 (BP2-BP3 or BP1-BP3) are recognised risk factors for developmental delay (DD) and autism spectrum disorders (ASD) [1–4]. More recently, these duplications were implicated as risk factors for schizophrenia (SZ) [5–8], however data are limited.

**Fig 1:**
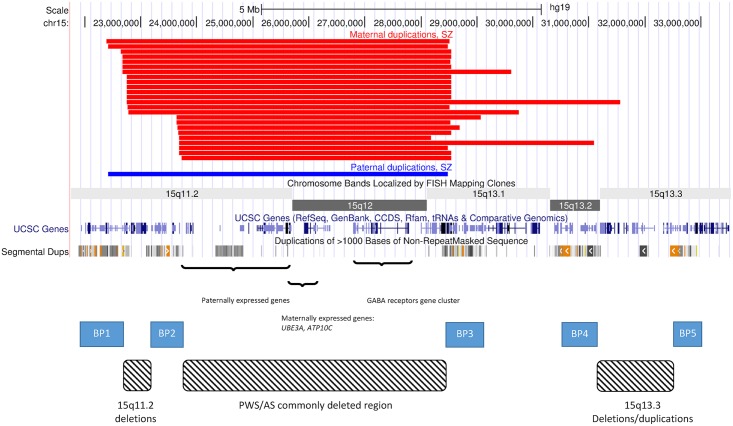
CNVs on chromosome 15. The image depicts the region on chromosome 15 that is affected by deletions and duplications caused by a number of low copy repeats. These form five recognised breakpoints (BPs) which cause the formation of deletions and duplications of different sizes. Several of them result in recognised syndromes: PWS/AS, 15q11.2 deletion and 15q13.3 deletion and duplication. The black bars at the top show the positions of the SZ/SZA probands in the current study (S1 Table). All four combinations of duplications between BP1 and BP4 are represented. They all intersect the regions of maternally and paternally expressed genes and the GABA receptors gene cluster.

The 15q11.2-q13.3 region contains a cluster of imprinted genes, which are expressed from one parental allele only as a consequence of germline epigenetic events (Fig 1). Within this cluster there are several paternally expressed genes, including *SNRPN*, *MKRN3*, *MAGEL2* and *NECDIN*, and two maternally expressed genes, namely *ATP10A*, and *UBE3A*. The genes within the 15q11.2-q13.3 interval are mostly canonical imprinted genes, in that expression is robustly monoallelic, although the imprinting status of *ATP10A* appears to be polymorphic and influenced by gender [9]. As a consequence of the presence of both maternally and paternally expressed imprinted genes, CNVs at this interval may be expected to have different phenotypes depending on their parent of origin. Indeed, most studies that have tested the parental origin of 15q11.2-q13.3 duplications show that those of maternal origin are usually responsible for the disease phenotypes. However, the penetrance of 15q11.2-q13.3 duplications has not yet been estimated. Moreover, whilst rare duplications of paternal origin have generally been regarded as benign [1,10], this has not been studied systematically. The assumption that duplications of paternal origin are benign comes from small numbers of observations of healthy mothers who carry duplications of paternal origin (and have transmitted them, making them maternal in the affected offspring), as well from the fact that they are much rarer in cohorts of DD/ASD children [1,10]. However, this pattern can also be explained by a lower penetrance of the paternal duplications and a lower prevalence in the general population. Only very large studies on patients and healthy controls can provide an accurate estimate of their role. The observations of paternal duplications occurring *de novo*, their extreme rarity in controls and their large size and high gene content, made us suspect that they are under selection pressure, like all other similar CNVs for which we have calculated the selection pressure [11] and therefore are likely to be pathogenic, although with a lower penetrance.

The aim of this study was to conduct the largest and most detailed assessment of 15q11.2-q13.3 interstitial duplications to date. By examining large cohorts of DD, ASD and SZ, along with large numbers of controls, we were able to estimate the prevalence, penetrance, and selection coefficients of 15q11.2-q13.3 interstitial duplications of maternal and paternal origin for SZ and for other neurodevelopmental disorders, and to identify clinical features common to SZ carriers of these duplications. We clearly implicate 15q11.2-q13.3 interstitial duplications of paternal origin in the aetiology of DD, but do not find them at increased rates in SZ, which is significantly associated only with duplications of maternal origin. These data clarify the contribution of imprinted genes within the PWS/AS interval to psychopathology, and have important, tangible benefits for patients with 15q11.2-q13.3 duplications by aiding genetic counseling.

## Results

### Prevalence of 15q11-q13 interstitial duplications

The prevalence rates of 15q11.2-q13.3 interstitial duplications in SZ, other neurodevelopmental disorders, and controls are shown in Table 1. Among 28,138 SZ probands, there were 25 individuals with 15q11.2-q13.3 interstitial duplications, of whom 24 were of maternal and only one of paternal origin. Prevalence estimates in SZ were therefore 0.085% (95%CI = 0.057–0.13%) for maternal and 0.0036% (95%CI = 0.00064–0.02%) for paternal duplications.

**Table 1:** Prevalence of 15q11-q13 duplications in disease and control cohorts.

Data Source	Country/Cohort	SZ		DD/ASD/MCA			Controls		Array
		Maternal	Paternal	Maternal	Paternal	Totals	Maternal	Paternal	
Costain et al. [7]	Canada	3/459	0/459				0/416	0/416	Affymetrix
Priebe et al. [12]	Germany	1/1,637	1/1,637				0/1,627	0/1,627	Illumina
Vacic et al. [13]	US/Ireland	0/802	0/802				0/742	0/742	NimbleGen
Szatkiewicz et al. [14]^a^	Sweden	0/4,097	0/4,097				0/5,480	0/5,480	Various
Ingason et al. [5]^b^	Europe (SGENE+ISC)	4/6,898	0/6,898				0/9,848	0/9,848	Various
See S1 Table^c^	Iceland	1/698	0/698	2/491ASD	0/491ASD	2/491	4/115,000	4/115,000	Illumina
See S1 Table	Germany	4/400	0/400				0/400	0/400	Illumina
Rees et al. [8]	UK: CLOZUK	8/6,882	0/6,882				0/11,255	0/11,255	Illumina
Ikeda et al, [15]	Japan	0/575	0/575				0/564	0/564	Affymetrix
See S1 Table	Japan	1/1,745	0/1,745				0/837	0/837	NimbleGen
Levinson et al. [16]	USA (MGS)	2/3,945	0/3,945				0/3,611	0/3,611	Affymetrix
Pinto et al. [17]	AGP, SSC, AGRE			10	2	13/5,106			Various
Ahn et al. [18] + S1 Table	UK (BBGRE)			7	2	20/20,260			Agilent
Urraca et al. [19]	Dup15q Alliance, USA			10	4	^d^			Various
Al Ageeli et al. [20]	France			10	1	^d^			Various
Dittwald et al. [21]	Baylor, USA					18/25,144			Agilent
Aypar et al [10]	Mayo, USA			11	1	^d^			Various
**Total**		**24/28,138**	**1/28,138**	**50**	**10**	**53/51,001**	**4/149,780**	**4/149,780**	
**Prevalence (95% CI)**		**0.085% (0.057–0.13%)**	**0.0036% (0.00064–0.02%)**	**0.087% (0.065–0.12%)**	**0.017% (0.009–0.033%)**	**0.1% (0.079–0.14%)**	**0.0027% (0.001–0.0069%)**	**0.0027% (0.001–0.0069%)**	

SZ; schizophrenia, DD; developmental delay; ASD; autism spectrum disorder, MCA; multiple congenital anomalies, SGENE; cohorts from European populations, described in Ingason et al. [5], ISC; International Schizophrenia Consortium, UK; United Kingdom, CLOZUK; patients from the UK treated with Clozapine, described in Rees et al. [8], MGS; Molecular Genetics of Schizophrenia Consortium, AGP; Autism Genome Project, SSC; Simon’s Simplex Collection, AGRE; Autism Genetics Research Exchange.

^a^ The total number of SZ subjects reported in the manuscript was 4,719; however 622 overlapped with the ISC publication and were removed.

^b^ Excludes individuals from Iceland who are analysed separately in this study, under the “Ingason (new data)^c^”

^c^ 115,000 people from Iceland, genotyped with microarrays have been included in the current study. These include the people presented in the Ingason et al. [5] study. As this is a population-based study, it is not practical or appropriate to select unrelated cases only, so we include carrier relatives as well.

^d^ The studies of Urraca et al. [19], Al Ageeli E et al. [20] and Aypar et al. [10], could not be used for estimating the prevalence of the duplications, as the overall numbers of patients tested were not specified. They are used for estimating the ratios between maternal and paternal duplications.

“new data” indicate cases/cohort analysed for the purpose of the current study

BBGRE: Brain & Body Genetic Resource Exchange, https://bbgre.brc.iop.kcl.ac.uk/, Ahn et al. [18]

Among 51,001 probands with DD/ASD/MCA from two large studies on referrals to clinical genetics clinics with DD/ASD/MCA [18,21], two from ASD cohorts and the current study [5,17], 53 (0.1%) had an interstitial 15q11.2-q13.3 duplication. Isodicentric chr15 (idic15) and interstitial triplications reported in these 51,001 probands (N = 6) were excluded from analysis, while no triplications were observed in SZ or control subjects. Only a small proportion of the previously published duplications had been reported for parental origin. In order to arrive at a more precise estimate of the ratio between maternal and paternal interstitial duplications in DD/ASD/MCA cohorts, we analysed an additional 13 individuals from the BBGRE database (20,260 individuals analysed for the current study) and one new ASD case from Iceland, and included 37 DD/ASD/MCA subjects from three studies that estimated the parental origin of duplications in such patients, but reported no population prevalence data [10,19,20] (Table 1). This gave us a total of 60 DD/ASD/MCA subjects with established parental origin: 50 (83.3%) maternal and 10 (16.7%) paternal. Using these proportions, we extrapolated the prevalence rates of maternal and paternal duplications for the 53 DD/ASD/MCA systematically ascertained carriers at 0.087% (95%CI = 0.065–0.12%) for maternal and 0.017% (95%CI = 0.009–0.033%) for paternal duplications, respectively (Table 1). Among 149,780 controls, there were four individuals with maternal and four with paternal duplication origin, giving identical rates of 0.0027% for both parental types (95%CI = 0.001–0.0069%).

Analysis of bipolar disorder (BD) datasets was not among the aims of this study. Just for the record, our review of 8,084 BD probands [22] found no 15q11.2-q13.3 duplication carriers. Although we reported one such case in our original publication [5], the available data suggests that these duplications do not play any significant role in BD.

### Penetrance of 15q11-q13 interstitial duplications

Table 2 shows the estimates for the prevalence of 15q11.2-q13.3 duplications in different population groups, and the estimated penetrance for SZ and other neurodevelopmental disorders. We estimated the general population frequency of 15q11.2-q13.3 duplications of maternal origin to be about two times higher than those of paternal origin (0.0069% vs. 0.0033%). The penetrance of maternal duplications for DD/ASD/MCA is very high (50.5%) and is about 2.5 times higher than those of paternal origin (20.7%). Maternal duplications also have high penetrance for SZ (12.3%) but in contrast, paternal duplications appear not to increase risk for SZ (penetrance of 1.1%), although we cannot completely exclude their role in SZ, as this estimate is based on a single observation in a SZ patient and therefore has large 95%CIs.

**Table 2:** Penetrance estimates for the 15q11.2-q13.3 duplication. In brackets are shown the 95%CI, calculated with the methods described in [24].

Parental origin of the 15q11-q13 duplication	Frequencies (%) (95%CI)				Estimated penetrance for SZ in %^a^	Estimated penetrance for DD/ASD/ MCA in %^a^
	Controls	SZ	DD/ASD/ MCA	General population^a^		
Maternal	0.0027 (0.001–0.0069)	0.085 (0.057–0.13)	0.087 (0.065–0.12)	0.0069 (0.004–0.013)	12.3 (4.5–31.6)	50.5 (20.5–100)
Paternal	0.0027 (0.001–0.0069)	0.0036 (0.00064–0.02)	0.017 (0.009–0.033)	0.0033 (0.0013–0.008)	1.1 (0.08–15.2)	20.7 (4.4–100)

^a^ Details for the methods used to calculate these estimates are presented in the Methods section.

**Fig 2:**
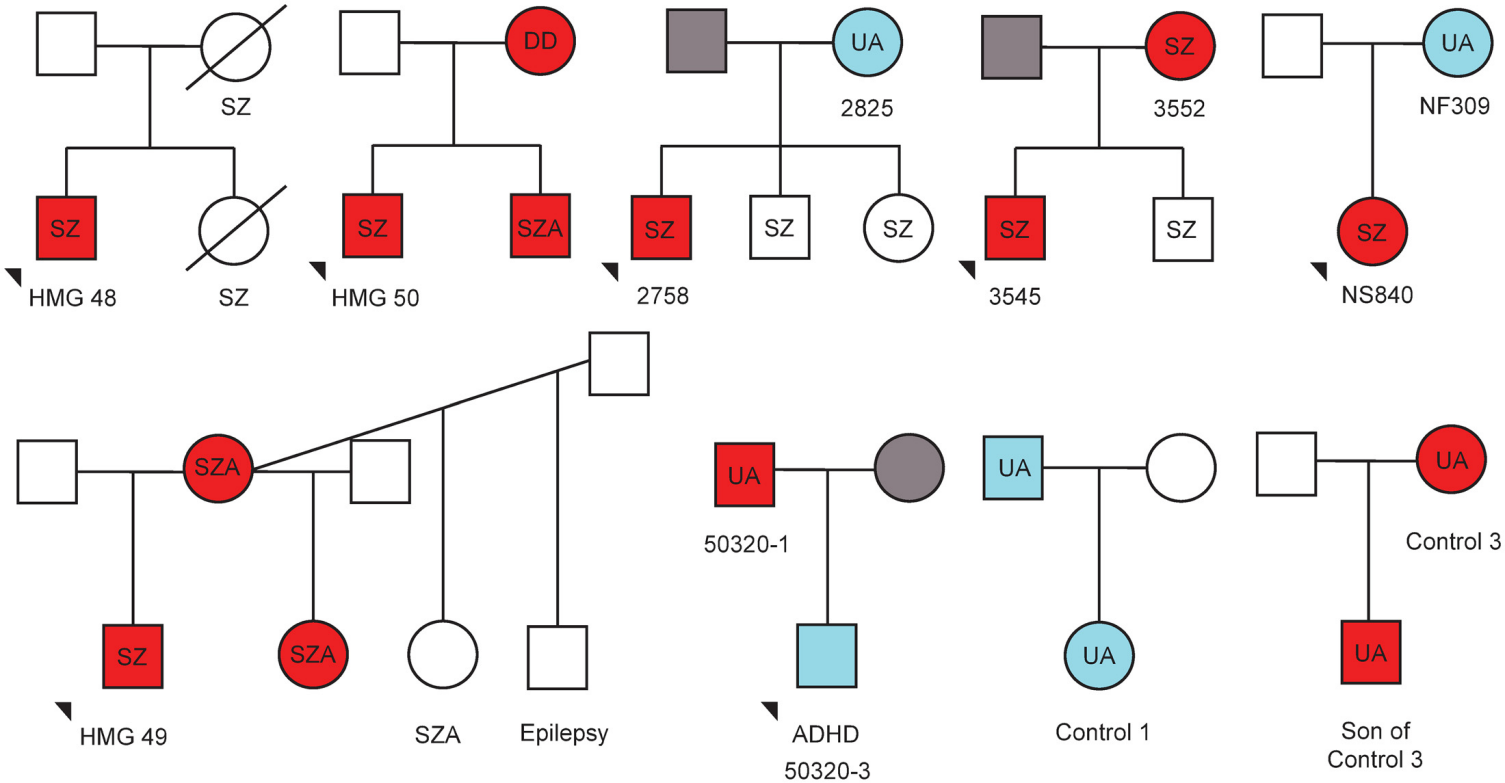
Family trees depicting transmission of 15q11.2-q13.3 duplications and neuropsychiatric phenotypes. Red fill indicates maternal duplications, blue indicates paternal duplications, and grey indicates no duplications. Samples where no DNA was available have no fill. Where DNA samples were available, parent of origin was determined using methylation-sensitive high-resolution melt curve analysis, or methylation-sensitive Southern Blot. Neuropsychiatric phenotype (detailed in S1 Table) is indicated as follows: SZ—schizophrenia; SZA—schizoaffective; DD—developmental delay; UA—unaffected. In addition, one individual was reported to have epilepsy and another ADHD.

We describe 10 relatives of probands that carried 15q11.2-q13.3 duplications (Fig 2 and S1 Table). Five of those were affected with psychosis or DD/ASD and all had 15q11.2-q13.3 duplications of maternal origin. Of the 5 unaffected relatives, only two had duplications of maternal origin (one of these, 50320–1, had depressive disorder). The three remaining unaffected individuals with paternal duplications had no reported neuropsychiatric phenotypes (but had not been specifically assessed). Six of the relatives were transmitting mothers and two were transmitting fathers. Five of the six mothers had offspring affected with SZ, while the two transmitting fathers had one healthy and one Attention Deficit Hyperactivity Disorder (ADHD) offspring (Fig 2). The remaining affected individuals in Fig 2, all marked with no fill (white), were not tested, as we had no DNA from them, but they are compatible with having maternal duplications.

### Selection coefficients

We do not have sufficient data on offspring of 15q11.2-q13.3 duplication carriers to make direct estimates of reproductive fitness and therefore present results for selection coefficients based on the ratio between *de novo* and inherited duplications: *de novo*/(*de novo*+inherited), following our previous work [11] (Table 3). The estimated selection coefficients appear similar: 0.55 for maternal and 0.58 for paternal duplications (but small numbers of paternal duplications preclude accurate estimates). Using the formula discussed in the Methods (*μ = qs*), we estimated the mutation rates as follows: maternal duplications, 0.000069×0.55 ≈ 1 per 27,000 newborns and paternal duplications, 0.000033×0.58 ≈ 1 per 50,000 newborns. Here we use population rates, rather than allele frequencies (*q*), and mutation rates per newborn, rather than per gamete (*μ*), as the formula suggests, simplifying the presentation. We point out that the estimates for both *s* and *μ* are less reliable compared to those for other CNVs [11], and that we refer to mutation rates in newborns, rather than in germ cells, which differ by orders of magnitude (Discussion).

**Table 3:** Selection coefficient estimates for 15q11.2-q13.3 duplications. Selection coefficients are approximated as the proportion of *de novo* CNV out of the total number of CNVs: *de novo*/(*de novo* + inherited).

Study	Maternal			Paternal		
	*De novo*	Inherited	*s*	*De novo*	Inherited	*s*
Urraca et al. [19]	9	1	0.9	2	2	0.5
Al Ageeli et al. [20]	6	3	0.67	0	1	0
Pinto et al. [17]	5	5	0.5	2	0	1
Aypar et al. [10]	3	3	0.5	0	0	n/a
Current study	6	12	0.33	3	2	0.6
**Total**	**29**	**24**	**0.55**	**7**	**5**	**0.58**

### Clinical characteristics

Our collaboration compiled a series of 29 duplication carriers with SZ or schizoaffective disorder (SZA), including the affected relatives (S1 and S2 Tables), of which only a small proportion have been reported before [5,7,8]. The 20 SZ/SZA cases with data on age at onset had a relatively early mean age at onset of 18.1 (SD = 6.9) years and in five of those the illness started during childhood (<13 years). Developmental and/or cognitive data were available for 21 cases and 76% of them had recorded learning/developmental problems. These ranged from mild ID (n = 3) to borderline ID (n = 8) with the rest having unspecified or nonverbal learning difficulties. The median IQ score was 75 (range 62–89) among the 11 cases with formal IQ tests. Specific psychiatric symptoms included catatonia (n = 6), disorganized behaviour (n = 5) and prominent antisocial traits (n = 5). Epilepsy was reported in only one case.

Among the 54 cases included in this study (S1 and S2 Tables), there were three carriers where ADHD was listed as a phenotype. One of these was of paternal origin, from a study that tested 727 children with ADHD [23]. The only case with a paternal duplication among the 12 cases in the study of Aypar et al. [10] also had ADHD. Still, these numbers are clearly too small to conclude that paternal duplications have a specific role in ADHD.

It should be noted that several of the population controls who carried duplications (all from Iceland) were not specifically subjected to formal neuropsychiatric assessment (S1 Table). However, they had not been registered as psychiatric patients and there was no information to indicate that they had developmental delay. Interestingly, two of the controls (control 2 and control 4) recently participated in a neuropsychiatric test battery project conducted in Iceland conducted by the Icelandic authors of the current paper. This revealed that Control 2 (paternal origin duplication) has an IQ of 100 and Global Assessment of Functioning (GAF) score of 81 and also is registered as having dyslexia, while Control 4 (maternal origin duplication) has an IQ of 84 and GAF score of 90. A third “unaffected” control (Control 3) had Alzheimer’s disease at the age of 64. These observations indicate that even population controls who do not suffer with our target diagnoses, could have some subtle cognitive phenotypes, highlighting the variable penetrance of this CNV.

Seventeen individuals analysed for this study (probands with SZ/SZA, their affected relatives and one control), had extended clinical/physical data available (S2 Table). Congenital anomalies were rare: one with cleft palate and one with cardiomegaly. Dysmorphic features were more common, recorded in 10 carriers, and included micro-, macro- or dolichocephaly, high palate, facial asymmetry and others. Six cases (35%) had urological conditions including urethral stricture, polyuria, urge incontinence, nephrectomy and recurrent urinary tract infections. Endocrinology problems included hypocalcemia, hypercholesterolemia, diabetes mellitus and hypothyroidism, however we cannot state whether they are more common than in any population of SZ patients, or had been caused by medication.

## Discussion

Maternal duplications of the PWS/AS critical region at chromosome 15q11.2-q13.3 are known to be pathogenic, causing DD and intellectual disability [24], and are also among the most common single genetic risk factors for ASD [1,25]. More recently they were also implicated as risk factors for SZ [5–8], although with a much lower estimated penetrance than for DD/ASD [26] and based on very few observations. In contrast, paternal duplications have not been considered to be pathogenic [1,10]. Here we conducted the largest and most detailed assessment of interstitial duplications at 15q11.2-q13.3 to date. We were able to estimate the prevalence, penetrance, and selection coefficients of 15q11.2-q13.3 duplications of maternal and paternal origin for SZ and other neurodevelopmental disorders, and to identify clinical features common to SZ carriers of these duplications. We clearly implicate 15q11.2-q13.3 duplications of paternal origin in the aetiology of DD, but not for SZ, where only maternal duplications increased risk.

Our data confirm that maternal interstitial duplications have a high penetrance (62.4%) for any neurodevelopmental disorder. Much of this was accounted for by the DD/ASD/MCA group, and only 12.3% by SZ (Table 2). This CNV is found in about 1:1176 patients suffering with SZ (95%CI = 1:769–1:1754). In contrast, paternal duplications do not appear to increase risk for SZ, (penetrance of 1.1%), suggesting that only maternal duplications have a specific effect on psychosis (although a small role for paternal duplications cannot be confidently excluded due to the wide confidence intervals). The importance of epigenetic status of duplications at this interval was further underlined by analysis of a number of families (Fig 2). Duplications in two unaffected mothers had a DNA-methylation pattern indicative of being paternally derived, whereas their offspring, who possessed a maternally derived duplication, suffered from psychotic illness.

Although they appear to have no role in SZ, we show for the first time that paternal duplications are pathogenic, increasing the risk for DD/ASD/MCA with a penetrance of 20.7% (95%CI = 4.4–100). One reason why paternal duplications have been regarded as non-pathogenic in the past is their rare occurrence in patients. Here we demonstrate that they are also rare in the general population as a whole, with prevalence rates of 0.0033% for paternal and 0.0069% for maternal duplications (Table 2). Their pathogenicity is supported by the strong selection pressure operating against them (*s* = 0.58, Table 3).

We tested if some of these results could be due to an overestimation of the population prevalence of DD/ASD/MCA or SZ, as those referred for testing or taking part in SZ studies might constitute a more severely affected sub-group. However, even if we use lower population rates of 0.5% for SZ and 2% for the combined group of ASD/DD/MCA, our penetrance estimates remain very high (S3 Table). The penetrance might even be higher, if we include subtle cognitive phenotypes, as described above for those population controls who took part in formal neurocognitive testing.

The question arises as to why paternal duplications are rarer than maternal ones in the general population, despite their lower pathogenicity. One explanation is a lower mutation rate in males. Indeed we estimate (with great approximation) that 1:27,000 and 1:50,000 newborns have a mutation that arose on maternal and paternal chromosomes, respectively. This refers to the rate in newborns with *de novo* mutations, while the mutation rate in sperm is much higher, observed in about 1:400 sperm cells [27], indicating a strong negative selection against such embryos before birth. Differences in the mutation rates according to the parental origin have been shown for other CNVs as well [28], so this may be one explanation of the observed difference.

The (possibly) lower rate of *de novo* mutations of paternal origin among newborns cannot, on their own, explain their extreme rarity in the population. Firstly, paternal duplications should be less efficiently eliminated from the population by negative selection pressure, due to their lower penetrance for neurodevelopmental disorders. Secondly, some maternal duplications will change to paternal when transmitted from male carriers. We now suggest one further explanation for their rarity: male patients with SZ and other neurodevelopmental disorders have lower fecundity. Indeed, men suffering with SZ have only half the number of offspring compared to women with SZ [29]. This effect was demonstrated for another high-penetrance CNV, the 22q11.2 deletion, where male carriers had on average three times fewer offspring than female carriers with the same deletion (0.3 vs. 0.9 per carrier) [30]. This parental bias could reduce substantially the number of inherited paternal 15q11-q13 duplications, compared to maternal ones.

We did not observe any trend for SZ patients to have a particular size of duplication, identifying individuals with all four possible combinations of breakpoints between BP1 and BP4 (Fig 1). Within the region common to all these duplications (BP2-BP3), the most likely candidate gene casing the neurodevelopmental phenotypes is *UBE3A* (Fig 1). It is only expressed from the maternal allele in neurons [31] and so maternal, but not paternal, duplications spanning this gene would lead to an over-dosage of expression in the brain. *ATP10A* is also maternally expressed, but the polymorphic nature of the imprinting of this gene [9] makes the canonically imprinted *UBE3A* the main causal candidate from an epigenetic perspective. Moreover, *UBE3A* also has a strong neural pedigree as it encodes E6-AP ubiquitin ligase, which is important in the degradation of proteins such as p53 and the ubiquitins [32], and has been shown to influence the glutamatergic system via its action on the synaptic protein Arc [33]. The role of this gene is supported by a recent clinical case with a micro-duplication encompassing only *UBE3A*, pointing to this gene as being key to the neuropsychiatric phenotypes [34].

The most consistent phenotypic characteristic of SZ carriers of these duplications is their cognitive deficit. Features of DD or intellectual deficit were recorded in 76% of carriers of maternal duplications on whom data was available. The median IQ score was 75 (range 62–89) among those who had a test (although this IQ result might be biased towards lower scores, as patients with more obvious learning problems are more likely to be referred for such testing). Congenital anomalies were fairly rare but mild dysmorphic features were observed in 10/17 cases with available clinical data.

The interest in the clinical presentation of psychosis among carriers of 15q11.2-q13.3 duplications of maternal origin started following reports that individuals with Prader-Willi syndrome who had maternal uniparental disomy (two maternally inherited copies and no paternal copy) might present with cycloid psychoses (acute polymorphic clinical pictures with prominent affective or motor symptoms) [35,36]. In our original paper [5] we noted that one of our cases had SZA and one had prominent affective symptoms. Here we present more cases with extended clinical descriptions. The rate of SZA disorder was 20% (4 out of 20) for cases with more detailed clinical records. SZ/SZA patients tended to have an early age at onset (mean of 18.1 years, SD = 6.9). Catatonia was recorded in six patients and disorganised or aggressive behaviour in eight patients. Antisocial traits were noted in all five cases from Canada. Although no single clinical picture emerges, it appears that psychotic patients with maternal 15q11.2-q13.3 duplications are more likely to present with disorganised, aggressive, antisocial and/or catatonic features. We did not find cases with cycloid psychoses, suggesting this may be restricted to Prader-Willi syndrome patients with maternal uniparental disomy as a consequence of the combined effect of both the increased dosage of maternally expressed genes, and the loss of paternally expressed genes.

We acknowledge certain limitations in our study. SNP arrays or array CGH can accurately determine extent and copy number of unbalanced chromosome regions (S2 Fig shows a selection of duplications and triplications tested with Illumina SNP arrays), but give no information on the structural arrangement and position of the material. This can only be established by examining chromosome preparations, in conjunction with FISH probes. As we only had DNA material, this was not possible for this study. However, the presence of three copies is generally indicative of an interstitial duplication, whilst four copies (triplication) suggests the presence of a supernumerary idic15. We excluded triplications from our study. Some rare idic15 cases can also represent only duplications and be indistinguishable from interstitial duplications by array testing. Therefore, while the vast majority of duplications in this study are likely to be interstitial, a small number could be supernumerary chromosomes, but still representing only duplications of the genetic material. As the main factor for pathogenicity is likely to be the copy number of the genes in the region, we feel that this limitation does not affect our conclusions. Interstitial triplications/idic15 are not the subject of our work, but we can report that there were no triplications in SZ subjects. This could be due to higher pathogenicity, leading to early onset neurodevelopmental phenotypes. Another limitation of our study concerns the role of paternal duplications in SZ, were we cannot completely exclude a small role, due to the wide confidence intervals for the prevalence and penetrance estimates. Finally, as discussed above, several unaffected controls had not had formal testing and although we can be confident that they do not suffer with severe neurodevelopmental disorders, we cannot exclude subtle phenotypes, and indeed have detected certain problems among the tested controls. This is not surprising, as a large study from Iceland has already shown that “healthy”carriers of pathogenic CNVs have lower cognitive performance [39].

In conclusion, our study clarifies the distinct roles of maternal and paternal interstitial duplications at 15q11.2-q13.3 in neuropsychiatric disorders, underlining the importance of maternally expressed imprinted genes in this interval to the incidence of psychotic illness. We also show that paternal duplications are pathogenic, increasing risk for DD/ASD/MCA with a penetrance of 20.7%. Defining the parent-of-origin of duplications at 15q11.2-q13.3, which does not require parental DNA, may allow the refinement of genetic counselling and/or therapeutic intervention for individuals carrying these CNVs.

## Methods

### Ethics statement

The CLOZUK study has UK National Research Ethics approval (Ethics Committee WALES REC 2, Study ID: 10/WSE02/15). The CLOZUK samples were collected anonymously from across the UK (thus without express consent), consistent with the UK Human Tissue Act and with the approval of the above ethics committee.

### Estimating the prevalence of 15q11.2-q13.3 duplications

We collated the available clinical and molecular data from large (>400 cases), systematically ascertained CNV studies of SZ from the literature, or known to us via our collaborations. We similarly collected data on other neurodevelopmental disorders such as DD/ASD and multiple congenital anomalies (MCA), focusing on two large studies based on referrals to genetic clinics and on three studies that specifically determined the parental origin of these duplications and reported on the presence of triplications/idic15 (Table 1). We used the reported information on parental origin of the 15q11.2-q13.3 duplications or, where possible, obtained DNA samples to establish this (details in S1 Text). Most teams that we approached responded to our requests, but some had no access to patient DNA and could not take part.

The control cohorts included 149,780 individuals (Table 1). The largest sample was from Iceland (~115,000 genotyped individuals at the time writing), a population-based cohort containing related individuals and those affected with medical and/or neuropsychiatric conditions. An ideal control cohort would only have one individual per family to avoid biases from over-sampling within population lineages. However, the Icelandic sample is designed to achieve total population coverage; therefore it is impractical to single out unrelated individuals. The rate of 15q11.2-q13.3 duplications in this population was assumed to be unbiased without excluding relatives of individuals with or without 15q11.2-q13.3 duplications. In fact, this population approaches the ideal general population ascertainment, that should give the best estimates for the various analyses performed here, therefore we decided to retain the two pairs of relatives who were carriers in this population.

### Estimating the penetrance of 15q11.2-q13.3 duplications

Penetrance was estimated according to the formula originally proposed by Vassos et al. [37], updated for the joint effect of DD/ASD/MCA and for SZ, as we proposed earlier [26]. Briefly, we first estimated the rate of the duplications in the general population. We assumed that the control (healthy) population comprises 95% of the population, as explained in our previous work [11]. The rest of the population is made up of approximately 1% SZ and 4% DD/ASD/MCA cases. From this we estimated the proportion of 15q11.2-q13.3 duplication carriers in the general population that develop SZ or DD/ASD/MCA (the penetrance). Although many duplication carriers might have both SZ and DD/ASD/MCA, we made the simplifying assumption that they would usually be ascertained only once. As the DD/ASD/MCA patients referred for genetic testing and the SZ patients recruited for studies might represent a more severely affected sub-group, we repeated the estimates after reducing by half these population rates (down to 0.5% for SZ and 2% for DD/ASD/MCA) and show these results in the S1 Text, S3 Table. Estimating 95% confidence intervals for the frequencies in each population followed the method we used in our previous paper [26]. Briefly, we first estimated the binomial CIs for the frequencies of CNVs in each population, including controls and the general population, using the Wilson score interval. Upper and lower 95% bounds for penetrance were estimated from the upper or lower bounds of CNV frequencies in patients and the lower or upper bounds of the frequencies in the general population.

### Selection coefficients

According to the mutation-selection balance theory, pathogenic mutations in the general population are found at low frequencies, where the addition of *de novo* mutations is balanced by the selection pressure against them (*q = μ/s*), where *q* is the allele frequency in the general population, *μ* is the mutation frequency and *s* is the selection coefficient [11,26]. In order to estimate the selection coefficients (*s*) of 15q11.2-q13.3 duplications of maternal and paternal origin, we used the methods outlined in our previous work [11,26] as follows: The selection coefficient of a CNV can be approximated as the proportion of *de novo* CNVs out of the total number of CNVs (*de novo* and inherited) that are observed in an unbiased sample of CNV carriers. This is because the number of CNVs filtered out by natural selection is approximately equal to those introduced in the population as *de novo* mutations, after making the simplifying assumption that the frequency of the CNV does not change from generation to generation. We then estimated the mutation rate of the duplications, using the above formula. We should point out that the actual differences between maternal and paternal mutation rates are prone to error, e.g. due to the flipping of maternal and paternal duplications according to the gender of transmitting parents and possible differences in fecundity between affected male and female carriers (Discussion).

### Establishing the parental origin

We established the parental origin of 15q11.2-q13.3 duplications for carriers from studies where it was previously unknown or not reported (Table 1 and S1 Table). These were the studies/datasets by Gawlick, Alexic, the BBGRE database [18] (https://bbgre.brc.iop.kcl.ac.uk/), and new carriers in the Icelandic population and three published datasets [12,16,23]. Parental origin was established by one of several methods. For DNA available to the Cardiff laboratory, we used a methylation-sensitive high-resolution melt curve analysis, as described previously [8,19,38], (S1 Text and S1 Fig). The same method was used for the samples from Canada [7]. In the Icelandic sample parental origin was either determined with methylation-sensitive Southern analysis [5] or by long-range haplotype analysis (S1 Text). Microsatellite analysis was used for the BBGRE dataset [18].

## Supporting Information

S1 Text Further details of sample sources and methodology. (DOC)

S1 Fig Illustrative example of methylation sensitive high-resolution melt-curve analysis of 15q11.2-q13.3 duplication CNV parent-of-origin. (PPTX)

S2 Fig Examples of B Allele Frequency and Log R Ratios (Illumina arrays) for 4 schizophrenia duplication carriers (top part of the figure) and 2 developmental delay triplication carriers for BP1-BP4 region (with duplicated BP4-BP5 region), demonstrating the unambiguous pattern of traces that distinguish duplications from triplications in the region: triplications have 5 bands of B Allele Frequency traces, while duplications have 4 bands. (DOC)

S1 Table Neuropsychiatric characteristics of subjects described in this study. Details of abbreviations can be found in the “Coding Key” tab.
(XLSX)

S2 Table Medical characteristics of subjects described in this study. Details of abbreviations can be found in the “Coding Key” tab.
(XLSX)

S3 Table Penetrance and population frequency estimates for the 15q11-q13 duplication, assuming lower population frequencies of SZ (0.5%) and DD/ASD/MCA (2%), and a resulting frequency of healthy controls of 97.5%. (DOCX)
