# Supplementary material for: Parental Origin of Interstitial Duplications at 15q11.2-q13.3 in Schizophrenia and Neurodevelopmental Disorders
Source: PLoS Genet. 2016 May 6;12(5):e1005993. doi: 10.1371/journal.pgen.1005993 (PMC4859484; doi:10.1371/journal.pgen.1005993)
Supplement: S1 Text — (DOC) [file pgen.1005993.s001.doc]

**Supplementary material**

1. **Sources of cases and controls where CNV carriers were identified**

**Germany**, Wuerzburg, M. Gawlick and G. Stoeber: This team tested a sample of 400 German parent-proband trios with schizophrenic psychoses with Illumina arrays, genotyped in the Cardiff laboratory. Putative CNVs were identified using PennCNV and validated with additional Illumina arrays. Duplications were found in 4 probands with schizophrenia. Two of them inherited them from mothers, (one healthy mother who herself had a paternal duplication, one with schizophrenia who herself carried a maternal duplication). The other two probands had *de novo* CNVs, both of maternal origin. All carriers of maternal duplications were affected with catatonic subforms of schizophrenia. All parental origin results were confirmed with methylation-sensitive PCR in the Cardiff laboratory (described in the main text and Supplementary material.).

**Germany**, Bonn/Munich, F. Degenhardt, D. Rujescu: The sample consists of 1,637 patients with SCZ or schizoaffective disorder and 1,627 controls, described in Priebe et al (2013). All individuals were genotyped on Illumina arrays and putative CNVs were identified using QuantiSNP and PennCNV. Two patients were carriers of the duplication. Parental origin was determined with methylation-sensitive PCR in the Cardiff laboratory. One had maternal and the other one a paternal duplication (the only paternal duplication in a SZ patient in the current study).

Priebe L, Degenhardt F, Strohmaier J, Breuer R, Herms S, Witt SH, Hoffmann P, Kulbida R, Mattheisen M, Moebus S, Meyer-Lindenberg A, Walter H, Mössner R, Nenadic I, Sauer H, Rujescu D, Maier W, Rietschel M, Nöthen MM, Cichon S. Copy number variants in German patients with schizophrenia. *PLoS One*. 2013 Jul 2;8(7):e64035.

**Japan**: Norio Ozaki, Branko Aleksic and Itaru Kushima. This team genotyped 1745 schizophrenia patients and 837 controls with NimbleGen 720K arrays. One proband had a duplication. Testing of her healthy mother revealed that she was also a carrier. The parental origin of the mother’s duplication was established with methylation-sensitive PCR in Cardiff (described in the main text and separate section in the Supplementary material). The healthy mother had a duplication of paternal origin.

**Iceland; Europe**, SGENE consortium, A. Ingason, T. Werge, H. Stefansson, K. Stefansson. In the original publication (Ingason et al 2011) we described five carriers who had schizophrenia among 7582 schizophrenia cases, and five controls, out of 41370 controls (the consortium samples were genotyped on a variety of arrays). There was also one bipolar and one ASD carrier. Of the controls, 3 had maternal and 2 had paternal duplications. All affected people had maternal duplications. 2792 controls (from the WTCCC dataset) overlapped with those from Rees et al (2014) and with other datasets, and are counted only once in the analysis (in the Rees et al, 2014 dataset). A new sample from the general population of Iceland is now added to the analysis, bringing the overall number of controls tested in Iceland to ~115,000. This identified one more control with paternal duplication and 2 healthy relatives of the controls described in the 2011 paper, (one with paternal, one with maternal duplications). One new ASD case had also a maternal duplication. The parental origin of the four newly discovered duplications was determined through comparison of chip genotypes from the probands with genotypes from their parents and/or siblings. When one or both parents were not genotyped, long range phased haplotypes were imputed from siblings or other relatives, (see method in Kong et al., 2008). After determining the parental genotypes either directly or through imputation, the team sought out markers within the duplicated segment for which the parents were homozygous for opposite alleles and that were directly typed in the proband. After that the team compared the B-allele frequencies at these markers in the proband and derived the call AAB if BAF<0.5 and ABB if BAF>0.5 and finally compared these calls with the parents’ genotypes. As the 15q11.2-q13.3 duplications always span at least several Mb they found for each proband more than enough loci, where the above specified criteria were met, to confidently derive the parent of origin. The Icelandic control population of 115,000 people is analysed here as a single one, combining the findings form the 2011 paper and the new results.

Ingason A, Kirov G, Giegling I, Hansen T, Isles AR, Jakobsen KD, Kristinsson KT, le Roux L, Gustafsson O, Craddock N, Möller HJ, McQuillin A, Muglia P, Cichon S, Rietschel M, Ophoff RA, Djurovic S, Andreassen OA, Pietiläinen OP, Peltonen L, Dempster E, Collier DA, St Clair D, Rasmussen HB, Glenthøj BY, Kiemeney LA, Franke B, Tosato S, Bonetto C, Saemundsen E, Hreidarsson SJ; GROUP Investigators, Nöthen MM, Gurling H, O'Donovan MC, Owen MJ, Sigurdsson E, Petursson H, Stefansson H, Rujescu D, Stefansson K, Werge T. Maternally derived microduplications at 15q11-q13: implication of imprinted genes in psychotic illness. *Am J Psychiatry*. 2011 Apr;168(4):408-17.

Kong A, Masson G, Frigge ML, Gylfason A, Zusmanovich P, Thorleifsson G, Olason PI, Ingason A, Steinberg S, Rafnar T, Sulem P, Mouy M, Jonsson F, Thorsteinsdottir U, Gudbjartsson DF, Stefansson H, Stefansson K: Detection of sharing by descent, long-range phasing and haplotype imputation. *Nat Genet* 2008;40: 1068-75.

**UK**, CLOZUK (Clozapine UK) sample, J. Walters, M.J. Owen: We analysed patients with schizophrenia (n = 6,882) and controls (n = 11,255), reported in Rees et al, (2014), all typed with Illumina arrays. Eight carriers were identified and all were found to have duplications of maternal origin when tested with methylation-sensitive PCR in the Cardiff laboratory.

Rees E, Walters JT, Georgieva L, Isles AR, Chambert KD, Richards AL, Mahoney-Davies G, Legge SE, Moran JL, McCarroll SA, O'Donovan MC, Owen MJ, Kirov G. [Analysis of copy number variations at 15 schizophrenia-associated loci.](http://www.ncbi.nlm.nih.gov/pubmed/24311552) *Br J Psychiatry*. 2014 Feb;204(2):108-14.

**Canada**, A. Bassett, C. Lowther, G Costain: This team tested a community-based cohort of 459 unrelated adults with schizophrenia with Affymetrix Genome-Wide Human SNP Array 6.0, described in Costain et al, (2013). Parental origin of 15q11–q13 duplications was determined from Multiplex Ligation-dependent Probe Amplification analysis to determine methylation status of the imprinted gene SNRPN. This confirmed all 3 duplications to be of maternal origin. The 3 probands and additional affected relatives, described for the purpose of the current study (Figure S1), had maternal duplications.

Costain G, Lionel AC, Merico D, Forsythe P, Russell K, Lowther C, Yuen T, Husted J, Stavropoulos DJ, Speevak M, Chow EW, Marshall CR, Scherer SW, Bassett AS. Pathogenic rare copy number variants in community-based schizophrenia suggest a potential role for clinical microarrays. *Hum Mol Genet*. 2013 Nov 15;22(22):4485-501.

**USA, MGS** **(Molecular Genetics of Schizophrenia),** P. Gejman, J. Duan, S. Simovic, D. Levinson, C. Leites: This team tested 3,945 subjects with schizophrenia or schizoaffective disorder (2,671 of European and 1,274 of African American ancestry) and 3,611 controls (2,648 of European and 963 of African American ancestry) Genotyping was performed with Affymetrix Genome-Wide Human SNP Array 6.0 and CNVs were called with the Birdsuite software package. Two subjects with duplications were found and confirmed with Illumina arrays in Cardiff: one African American and one European American. Parental origin was tested in the Cardiff laboratory with methylation-sensitive PCR which showed that both had maternal duplications.

Levinson DF, Duan J, Oh S, Wang K, Sanders AR, Shi J, Zhang N, Mowry BJ, Olincy A, Amin F, Cloninger CR, Silverman JM, Buccola NG, Byerley WF, Black DW, Kendler KS, Freedman R, Dudbridge F, Pe'er I, Hakonarson H, Bergen SE, Fanous AH, Holmans PA, Gejman PV. Copy number variants in schizophrenia: confirmation of five previous findings and new evidence for 3q29 microdeletions and VIPR2 duplications. *Am J Psychiatry*. 2011 Mar;168(3):302-16.

**Datasets without 15q11.2-q13.3 CNVs:**

**Japan,** M. Ikeda and N. Iwata: No duplications were found in 575 schizophrenic patients and 564 controls, genotyped with Affymetrix 6.0 arrays and called with the Birdsuite software package.

Ikeda M, Aleksic B, Kirov G, Kinoshita Y, Yamanouchi Y, Kitajima T, Kawashima K, Okochi T, Kishi T, Zaharieva I, Owen MJ, O'Donovan MC, Ozaki N, Iwata N. Copy number variation in schizophrenia in the Japanese population. *Biol Psychiatry*. 2010 Feb 1;67(3):283-286.

**US/Ireland**: Vacic 2011: tested 802 SZ patients and 742 controls using NimbleGen HD2 arrays and reported no such duplications.

Vacic V, McCarthy S, Malhotra D, Murray F, Chou HH, Peoples A, Makarov V, Yoon S, Bhandari A, Corominas R, Iakoucheva LM, Krastoshevsky O, Krause V, Larach-Walters V, Welsh DK, Craig D, Kelsoe JR, Gershon ES, Leal SM, Dell Aquila M, Morris DW, Gill M, Corvin A, Insel PA, McClellan J, King MC, Karayiorgou M, Levy DL, DeLisi LE, Sebat J. Duplications of the neuropeptide receptor gene VIPR2 confer significant risk for schizophrenia. *Nature*. 2011 Mar 24;471(7339):499-503.

**Sweden**, Szatkiewicz et al, 2014: This team tested 4,719 cases and 5,917 controls from Sweden. Genotyping was done in six batches (Sw1–6) using Affymetrix 5.0 (3.9%, Sw1), Affymetrix 6.0 (38.6%, Sw2–4), and Illumina OmniExpress (57.4%, Sw5–6). Genotypes were called using Birdsuite for Affymetrix or BeadStudio for Illumina arrays. After exclusion of overlapping samples with the ISC study (622 cases and 437 controls from this study were already included in Ingason et al, 2011), there were 4,097 cases and 5,480 controls independent of other datasets, who were included in the analysis.

Szatkiewicz JP, O'Dushlaine C, Chen G, Chambert K, Moran JL, Neale BM, Fromer M, Ruderfer D, Akterin S, Bergen SE, Kähler A, Magnusson PK, Kim Y, Crowley JJ, Rees E, Kirov G, O'Donovan MC, Owen MJ, Walters J, Scolnick E, Sklar P, Purcell S, Hultman CM, McCarroll SA, Sullivan PF. Copy number variation in schizophrenia in Sweden. *Mol Psychiatry*. 2014 Jul;19(7):762-73.

**Data on Autism and other early-onset neurodevelopmental disorders**:

**France**, Al Ageeli, et al: This is a study of 30 unrelated patients who were identified among the patients seen at the genetic clinics of Robert DEBRE hospital (general referral for genetic testing for developmental delay, speech retardation, epilepsy and/or autistic behaviour) with duplications of the 15q11.2-q13.3 region. Age ranged from 4 months to 30 years at the last evaluation (mean 8.8, median 7). The team used several methods to detect the duplications, including FISH.

There were 15 supernumerary marker chromosome 15 (SMC15). There were not considered for this study. We analysed the remaining 15 interstitial duplications, of which 10 were maternal, one paternal the origin of the others were not determined. 8 were de novo, 3 transmitted from mothers one from a father.

Al Ageeli E, Drunat S, Delanoë C, Perrin L, Baumann C, Capri Y, Fabre-Teste J, Aboura A, Dupont C, Auvin S, El Khattabi L, Chantereau D, Moncla A, Tabet AC, Verloes A. Duplication of the 15q11-q13 region: clinical and genetic study of 30 new cases. *Eur J Med Genet*. 2014 Jan;57(1):5-14.

**USA,** N. Urraca et al: Subjects were recruited through the Dup15q Alliance (http://www.dup15q.org), a family support group for individuals with chromosome 15 duplications. The array platforms used were either a targeted Signature-ChipOS array (*n* = 9) or Affymetrix 6.0 (*n* = 5); The team found 10 maternal duplications (all had ASD); 4 paternal ones: (50% had ASD). Parental origin was tested with the method used in the current study. *De novo* status was determined in all cases: 9 of 10 maternal duplications were *de novo*, as were 2 of 4 paternal ones.

Urraca N, Cleary J, Brewer V, Pivnick EK, McVicar K, Thibert RL, Schanen NC, Esmer C, Lamport D, Reiter LT. The interstitial duplication 15q11.2-q13 syndrome includes autism, mild facial anomalies and a characteristic EEG signature. *Autism Res*. 2013 Aug;6(4):268-79.

**North America + Europe,** D. Pinto et al, Autism Genome Project: Genotyped on Illumina 1M arrays 2,845 families and 2,640 control individuals of European ancestry. The team also reported in the Supplementary data on summary findings from the three largest datasets on autism spectrum disorders: Autism Genome Project (their own study), Simon Simplex Collection and AGRE. The total number of patients was 5,106, with 13 interstitial duplication carriers, 8 of which were de novo. 10 duplications were of maternal and 2 of paternal origin.

Pinto D, Delaby E, Merico D, Barbosa M, Merikangas A, Klei L, Thiruvahindrapuram B, Xu X, Ziman R, Wang Z, Vorstman JA, Thompson A, Regan R, Pilorge M, Pellecchia G, Pagnamenta AT, Oliveira B, Marshall CR, Magalhaes TR, Lowe JK, Howe JL, Griswold AJ, Gilbert J, Duketis E, Dombroski BA, De Jonge MV, Cuccaro M, Crawford EL, Correia CT, Conroy J, Conceição IC, Chiocchetti AG, Casey JP, Cai G, Cabrol C, Bolshakova N, Bacchelli E, Anney R, Gallinger S, Cotterchio M, Casey G, Zwaigenbaum L, Wittemeyer K, Wing K, Wallace S, van Engeland H, Tryfon A, Thomson S, Soorya L, Rogé B, Roberts W, Poustka F, Mouga S, Minshew N, McInnes LA, McGrew SG, Lord C, Leboyer M, Le Couteur AS, Kolevzon A, Jiménez González P, Jacob S, Holt R, Guter S, Green J, Green A, Gillberg C, Fernandez BA, Duque F, Delorme R, Dawson G, Chaste P, Café C, Brennan S, Bourgeron T, Bolton PF, Bölte S, Bernier R, Baird G, Bailey AJ, Anagnostou E, Almeida J, Wijsman EM, Vieland VJ, Vicente AM, Schellenberg GD, Pericak-Vance M, Paterson AD, Parr JR, Oliveira G, Nurnberger JI, Monaco AP, Maestrini E, Klauck SM, Hakonarson H, Haines JL, Geschwind DH, Freitag CM, Folstein SE, Ennis S, Coon H, Battaglia A, Szatmari P, Sutcliffe JS, Hallmayer J, Gill M, Cook EH, Buxbaum JD, Devlin B, Gallagher L, Betancur C, Scherer SW. Convergence of genes and cellular pathways dysregulated in autism spectrum disorders. *Am J Hum Genet*. 2014 May 1;94(5):677-94

**London, UK**, BBGRE database, C. Ogilvie & J.W. Ahn:

This dataset is from the Brain and Body Genetic Resource Exchange database (BB-GRE; [http://bbgre.org](http://bbgre.org/)), methodology described in Ahn et al, (2013); Tropeano et al, (2013); Cafferkey et al, (2014). The overall ethnic distribution (self-reported data) was 70% Caucasians, 15% Africans and 15% other/mixed ancestry. For the purpose of this study, the team analysed the data on 20,260 individuals with a range of neurodevelopmental conditions, clinically referred to Guy's & St Thomas Hospitals, London, for array comparative genomic hybridisation (aCGH) using a 60 K Agilent array to hybridise DNA extracted from blood. The results were analysed using Feature Extraction and Genomic Workbench (Agilent) in order to quantify the images and detect CNVs. Agilent ADM-2 algorithm at threshold 6 (with a 3 probe sliding window providing a median detection of 120 kb) was used to call CNVs.

We excluded from analysis 6 individuals with triplications/idic15. There were 20 individuals with interstitial duplications at the PWS/AS locus and on 11 of those the parental origin had been determined (3 paternal, 8 maternal). Microsatellite markers were used to check parental carrier status and parent of origin. Of the 12 patients where inheritance/*de novo* status was determined, there were 6 with *de novos* (2 paternal, 3 maternal, 1 unknown) and 6 with inherited duplications (5 from mothers, 1 from father). For the estimates of parental origin in the current analysis we only include 9 carriers with confirmed parental origin and who do not have a significant second CNV that could explain the phenotype (Table S1).

Cafferkey M, Ahn JW, Flinter F, Ogilvie C. Phenotypic features in patients with 15q11.2(BP1-BP2) deletion: Further delineation of an emerging syndrome. *Am J Med Genet* A. 2014 Aug;164(8):1916-22.

Tropeano M, Ahn JW, Dobson RJ, Breen G, Rucker J, Dixit A, Pal DK, McGuffin P, Farmer A, White PS, Andrieux J, Vassos E, Ogilvie CM, Curran S, Collier DA. [Male-biased autosomal effect of 16p13.11 copy number variation in neurodevelopmental disorders.](http://www.ncbi.nlm.nih.gov/pubmed/23637818) *PLoS One.* 2013 Apr 18;8(4):e61365.

Ahn JW1, Dixit A, Johnston C, Ogilvie CM, Collier DA, Curran S, Dobson RJ. BBGRE: brain and body genetic resource exchange. *Database (Oxford)*. 2013 Sep 27;2013:bat067.

**ADHD** **dataset** **from the UK**, J. Martin: The team (From Cardiff) tested 727 ADHD children with Illumina Human660W-Quad arrays (Stergiakouli et al, 2012), and found one carrier. Parents were available and inheritance was tested specifically for the current project. It was transmitted from the healthy father (i.e. the proband had a paternal duplication), while the father had a duplication of maternal origin. This was determined by methylation-sensitive PCR in the Cardiff laboratory.

Stergiakouli E, Hamshere M, Holmans P, Langley K, Zaharieva I; deCODE Genetics; Psychiatric GWAS Consortium, Hawi Z, Kent L, Gill M, Williams N, Owen MJ, O'Donovan M, Thapar A. Investigating the contribution of common genetic variants to the risk and pathogenesis of ADHD. *Am J Psychiatry*. 2012 Feb;169(2):186-94.

**USA, Baylor College**, P. Dittwald et al. The team tested the database at the Medical Genetics Laboratories of Baylor College of Medicine and checked once again for triplications for the purpose of the current study: 25,144 patients had been referred for genetic testing and subjected to Agilent genome-wide assays. All 18 cases reported in the paper had duplications (one further case had a triplication, but had been excluded from analysis in the original study, and of course, from the current study). Parental status was not analysed. This study is used to establish the rate of these duplications in subjects referred for genetic testing but not for parental origin.

Dittwald P, Gambin T, Szafranski P, Li J, Amato S, Divon MY, Rodríguez Rojas LX, Elton LE, Scott DA, Schaaf CP, Torres-Martinez W, Stevens AK, Rosenfeld JA, Agadi S, Francis D, Kang SH, Breman A, Lalani SR, Bacino CA, Bi W, Milosavljevic A, Beaudet AL, Patel A, Shaw CA, Lupski JR, Gambin A, Cheung SW, Stankiewicz P. NAHR-mediated copy-number variants in a clinical population: mechanistic insights into both genomic disorders and Mendelizing traits. Genome Res. 2013 Sep;23(9):1395-409.

**USA, Mayo clinic, Aypar et al.** A total of 35 cases with multiple copies of the PW/AS critical region were ascertained through the results database of the Cytogenetics Laboratory at the Mayo Clinic, USA. Array CGH was performed on three array platforms: Signature Genomics BAC, Agilent 44 k and 180 k arrays. Methylation-sensitive multiplex ligation-dependent probe amplification was used to determine the parent of origin of the extra copies. Cases of idic[15], ring[15] or tandem triplications were excluded from analysis in the current study. This left 11 maternal and 1 paternal duplication. 3 maternal duplications were *de novo*, 3 were maternally inherited, while the inheritance status of the remaining interstitial duplications was not established.

Aypar, U., Brodersen, P.R., Lundquist, P.A., Dawson, D.B., Thorland, E.C. and Hoppman, N. (2014) Does parent of origin matter? Methylation studies should be performed on patients with multiple copies of the Prader-Willi/Angelman syndrome critical region. *Am. J. Med. Genet. A,* **164A**, 2514-2520.

1. **Establishing the parental origin of duplications with methylation sensitive high-resolution melt-curve (HRM curve) analysis**

Where DNA was made available to the Cardiff laboratory, we used a methylation sensitive high-resolution melt-curve (HRM curve) analysis, as described by Urraca et al, (2010). The method exploits the methylation changes at the small nuclear ribonucleoprotein polypeptide N (SNRPN) promoter, which are parent-of-origin specific. This method can determine the parent-of-origin of this duplication without the need to have DNA from the parents. DNA samples were first subjected to bisulfite conversion using the EZ methylation kit Gold (Cambridge Bioscience,Cambridge, UK), following the manufacturer’s protocols. These bisulfite treated samples were then amplified using the Sensimix HRM kit (Bioline, UK) and the PCR product solution was analysed using dedicated HRM software (Rotor-Gene Q series software). Normalisation regions for the leading/trailing ranges were set at 73-79/86-93 ^o^C.

The melting curve analysis assigned unambiguously the parental status of duplications, (S1 Figure). The figure shows the results of unaffected controls (with no duplications, i.e. one maternal and one paternal copy), unaffected mothers carriers (with two paternal copies and one maternal one) and affected individuals with maternal duplications (two maternal and one paternal copy).

Urraca N, Davis L, Cook EH, Jr, Schanen NC, Reiter LT. A single-tube quantitative high-resolution melting curve method for parent-of-origin determination of 15q duplications. *Genetic Testing Mol Biomarkers*. (2010); **14**:571–576.
