# Supplementary material for: Parental Origin of Interstitial Duplications at 15q11.2-q13.3 in Schizophrenia and Neurodevelopmental Disorders
Source: PLoS Genet. 2016 May 6;12(5):e1005993. doi: 10.1371/journal.pgen.1005993 (PMC4859484; doi:10.1371/journal.pgen.1005993)
Supplement: S1 Fig — (PPTX) [file pgen.1005993.s002.pptx]

## Slide 1
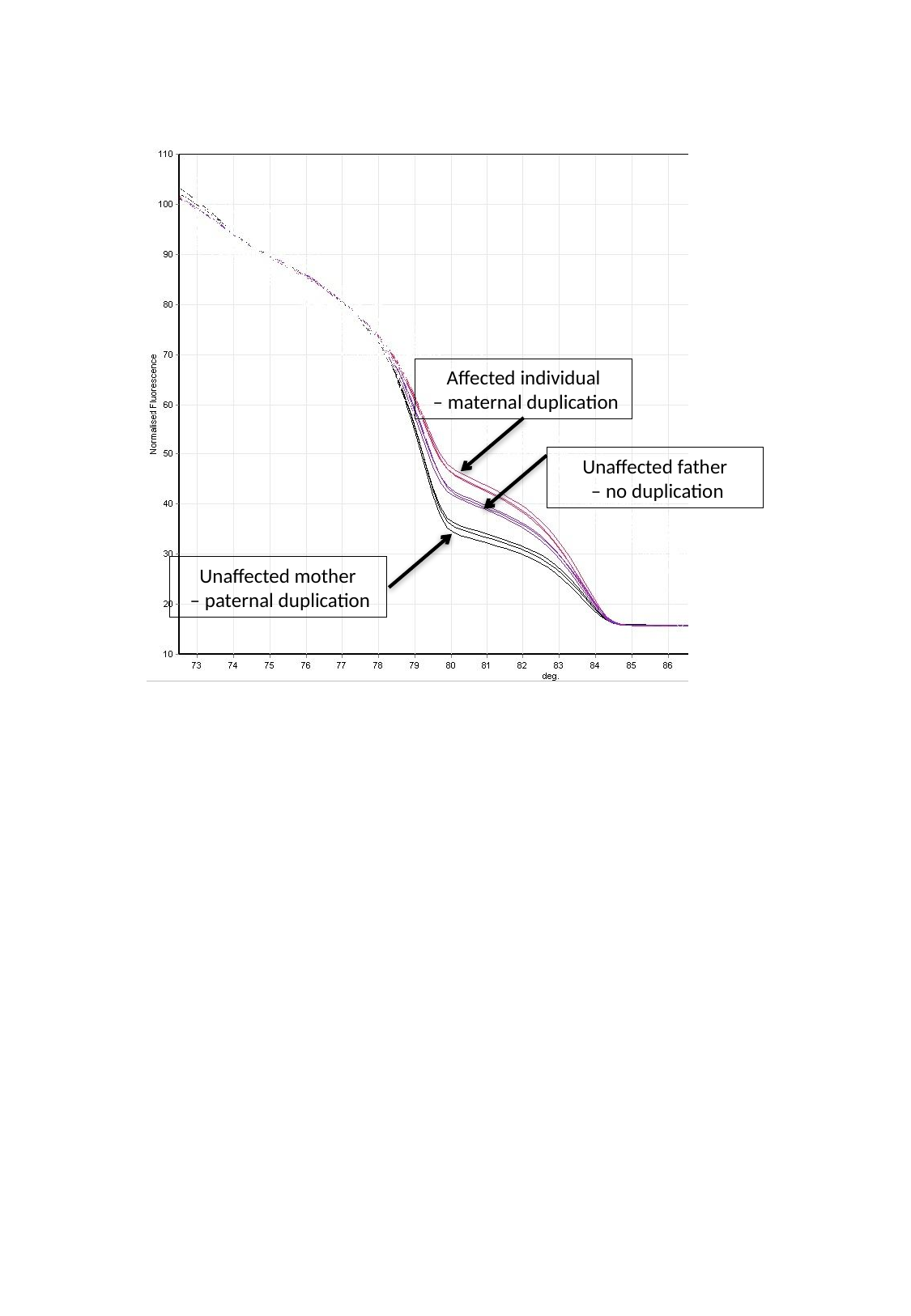

Affected individual
 – maternal duplication
Unaffected father
 – no duplication
Unaffected mother
 – paternal duplication
