# Supplementary material for: Parental Origin of Interstitial Duplications at 15q11.2-q13.3 in Schizophrenia and Neurodevelopmental Disorders
Source: PLoS Genet. 2016 May 6;12(5):e1005993. doi: 10.1371/journal.pgen.1005993 (PMC4859484; doi:10.1371/journal.pgen.1005993)
Supplement: S2 Fig — (DOC) [file pgen.1005993.s003.doc]

**Supplementary Figure 2**. Examples of B Allele Frequency and Log R Ratios (Illumina arrays) for 4 schizophrenia duplication carriers (top part of the figure) and 2 developmental delay triplication carriers for BP1-BP4 region (with duplicated BP4-BP5 region), demonstrating the unambiguous pattern of traces that distinguish duplications from triplications in the region: triplications have 5 bands of B Allele Frequency traces, while duplications have 4 bands.
