# Supplementary material for: Parental Origin of Interstitial Duplications at 15q11.2-q13.3 in Schizophrenia and Neurodevelopmental Disorders
Source: PLoS Genet. 2016 May 6;12(5):e1005993. doi: 10.1371/journal.pgen.1005993 (PMC4859484; doi:10.1371/journal.pgen.1005993)
Supplement: S3 Table — (DOCX) [file pgen.1005993.s006.docx]

| Parental origin of the 15q11-q13 duplication | Frequencies (%) | | | | Estimated penetrance for SZ in %^a^  (95% CI)^b^ | Estimated penetrance for DD/ASD/ MCA in %^a^  (95% CI)^b^ |
| --- | --- | --- | --- | --- | --- | --- |
|  | Controls  (95%CI) | SZ (95%CI) | DD/ASD/ MCA (95%CI) | General population^a^  (95%CI) |  |  |
| Maternal | 0.0027  (0.001-0.0069) | 0.085  (0.057-0.13) | 0.087  (0.065-0.12) | 0.0048  (0.0026-0.0098) | 8.9  (3-25.4) | 35.4  (13.3-93.8) |
| Paternal | 0.0027  (0.001-0.0069) | 0.0036  (0.00064-0.02) | 0.017  (0.009-0.033) | 0.003  (0.0011-0.0075) | 0.6  (0.04-8.6) | 11.4  (2.35-57) |

**Table S3.** Penetrance and population frequency estimates for the 15q11.2-q13.3 duplication, assuming lower population frequencies of SZ (0.5%) and DD/ASD/MCA (2%), and a resulting frequency of healthy controls of 97.5%.
